# Combining FRET and optical tweezers to study RhoGTPases spatio-temporal dynamics upon local stimulation

**DOI:** 10.14440/jbm.2017.159

**Published:** 2017-03-07

**Authors:** Federico Iseppon, Luisa MR Napolitano, Vincent Torre, Dan Cojoc

**Affiliations:** ^1^Neuroscience Area, International School for Advanced Studies, Trieste, Italy; ^2^Optical Manipulation Lab, CNR-IOM, the National Research Council of Italy - Institute of Materials, Trieste, Italy

**Keywords:** FRET, growth cones, guidance molecules, local stimulation, optical tweezers

## Abstract

Local stimulation with optical tweezers has been used to mimic natural stimuli that occur in biological processes such as cell migration or differentiation. Carriers (beads and lipid vesicles) with sizes down to 30 nm can be manipulated with a high spatial and temporal resolution: they are positioned with a sub-micrometric precision on a specific cell compartment and the beginning of the stimulation can be triggered with millisecond precision. RhoGTPases are a Ras-related family of proteins that regulate many different functions including cell polarity, microtubule dynamics and membrane transport pathways. Here we combine local stimulation with FRET microscopy to study RhoGTPases spatial and temporal activation following guidance cue local stimulation. We used two different vectors for local delivery: silica micro-beads and micro-sized lipid vesicles. The experimental methods associated with neuronal growth cone local stimulation are discussed in detail, as well as the analysis methods. Here we present a protocol that enables to study neuronal growth cone cytoskeleton rearrangements in response to a gradient of molecules in a way that better mimics physiological conditions, and it can be similarly applied to each secreted molecule involved in cell signaling.

## BACKGROUND

RhoGTPases are a Ras-related family of proteins that play a major role in many physiological processes including cell polarity, microtubule dynamics, membrane transport pathways and transcription factor activity. They act as molecular switches that cycle between two conformational states: one bound to GTP (“active” state); the other one bound to GDP (“inactive” state) [1]. In the activated form, they bind different effector proteins leading to the activation of myriad downstream signals [2]. The best-characterized function of Rho GTPases is in the regulation of F-actin dynamics in both non-neuronal cells and neuronal growth cones [3,4]. During axon guidance, RhoGTPases translate signals from extracellular cues into growth-cone movement and axon guidance: RhoA increases the acto-myosin contractility, whereas Rac1 and Cdc42 induce the polymerization of actin to form lamellipodial and filopodial protrusions, respectively [5,6]. RhoGTPases’ ability to regulate so many different functions, in highly dynamic cellular contexts, implies that they are tightly regulated at the spatio-temporal level. This has led to the development of fluorescent probes for the direct monitoring of their activation state in living cells based on the principle of Förster resonance energy transfer (FRET) [7-10]. Genetically encoded biosensors using protein-based affinity reagents and readout modules have been designed as intra-molecular (donor and acceptor on a single chain) [8] or inter-molecular (dual chain) [9] probes. FRET is an excellent readout for biosensors because small changes in the distance or orientation between the two fluorophores can cause large changes in FRET efficiency, allowing sensitive detection of protein binding or conformational changes. Measurement of FRET efficiency *via* ratiometric analysis, relying on the differences between the localization and activation images, is effective using intra-molecular probes. On the other hand, the use of intermolecular FRET design enhances sensitivity because, unlike the single chain design, there is no FRET when the biosensor is in the “off” state. The main disadvantage of the inter-molecular probes is that the two chains do not distribute equally through the cell, necessitating careful bleed-through corrections.

Most of the *in vitro* neuron stimulation experiments employ bath or micropipette administration of molecules to change the cell environment [11]. Hence, new strategies to address specific compartments of neuronal cells with spatial and temporal gradients of molecules, which better reflect cellular physiological conditions, should be considered [12]. Focal stimulation of neurons using optical tweezers and single micro-beads functionalized with a secretory molecule, the neurotrophin brain-derived neurotrophic factor (BDNF), demonstrated to induce focal increase of calcium signaling in the stimulated dendrite, with a specific activation of the TrkB receptor pathway and influenced the development of growth cones [13]. An elegant demonstration of the role of molecular gradients for cell stimulation has been reported for neutrophils by using optically manipulated microsources releasing soluble molecules that act as chemoattractants or perturb the actin cytoskeleton [14]. Another type of optically manipulated carriers are the lipid micro-vesicles encapsulating active molecules that are released by photolysis of the vesicle membrane using a laser pulse [15]. An optimization of this technique allowed the study of the effect of a very small number of different guidance cues on hippocampal neurons [16]. In principle, the number of molecules can be scaled down to a single molecule for a micron size vesicle at 1 nM concentration. Stimulation can be triggered with very high temporal precision, providing steep gradients over length scales from sub-micrometer to tens of micrometers and at time-scales from milliseconds to seconds.

Here, we present an experimental protocol that combines FRET microscopy with optical tweezers manipulation to observe the spatial and temporal activation of RhoA and Cdc42 following localized cell stimulation with Sema3A, a negative secreted guidance cue that steers a growth cone by inducing partial or local collapse [17,18]. We used NG108-15 neuroblastoma cells and an intermolecular RhoA/Cdc42 FRET sensor [19] for FRET. Two different vectors were used for Sema3A local delivery to specific sites of the cell through optical tweezers manipulation: silica micro-beads and micron-sized lipid vesicles. Combining FRET with local stimulation provides a new tool for the study of the cytoskeleton rearrangements in response to guidance cues stimulation, underlying the dynamic spatial and temporal activation of RhoGTPases.

## MATERIALS

### Cells

1. NG108-15 Neuroblastoma cell line (Sigma-Aldrich, cat # 88112302)

### Reagents

• Bicinchoninic Acid Protein Assay Kit (Sigma-Aldrich, cat # BCA1)
• BioMag Plus Carboxyl Coupling Kit (Bangs Laboratories Inc., cat # BP611)
• CaCl_2_ (Sigma-Aldrich, cat # C1016)
• DMEM (Thermo Fisher Scientific, cat # 31966-021)
• Ethanol (Sigma-Aldrich, cat # 02860)
• Fetal Bovine Serum (FBS) (Sigma-Aldrich, cat # F6178)
• Glass Coverslips (12 mm round) (Chemglass, cat # 89167-106)
• Glucose (Sigma-Aldrich, cat # G0350500)
• HCl (Sigma-Aldrich, cat # 318949)
• HEPES (Sigma-Aldrich, cat # H3375)
• KCl (Sigma-Aldrich, cat #P9333)
• Laminin from Engelbreth-Holm-Swarm murine sarcoma basement membrane (Sigma-Aldrich, cat # L2020)
• Liposome Kit (Sigma-Aldrich, cat # L4395)
• mCherry-Pak3(60-113)/S74A/F84A-mCherry-C1 (Addgene, plasmid # 29676)
• mCherry-Rhotekin(8-89)-mCherry-C1 (Addgene, plasmid # 29675)
• mEGFP-Cdc42-C1 (Addgene, plasmid # 29673)
• mEGFP-RhoA-C1 (Addgene, plasmid # 29674)
• Metafectene Easy + transfection reagent (Biontex Laboratories, cat # T090)
• MgCl_2_ (Sigma-Aldrich, cat # M8266)
• NaCl (Sigma-Aldrich, cat # S9888)
• PBS 10X, pH 7.4 (Thermo Fisher Scientific, cat # 70011044)
• Penicillin/Streptomycin (Biochrom AG, cat # A2213)
• Sucrose (Sigma-Aldrich, cat # S0389)

### Recipes

• NG108-15 culture medium: Add FBS and Penicillin-Streptomycin to respectively 10% and 1% of volume of DMEM. Store at 4^°^C for up to 8 weeks.
• Ringer Solution: Add NaCl 145 mM, KCl 3 mM, CaCl_2_ 1.5 mM, MgCl_2_ 1 mM, Glucose 5 mM, HEPES10 mM to 1L of MilliQ Water. Adjust the pH to 7.4.

### Equipment

• Cell culture incubator (humidified, 5% CO_2_)
• Biological hood with laminar flow
• Cell counter chamber
• Nikon Eclipse inverted fluorescence microscope (Nikon)
• IR laser 1064 nm (IPG photonics)
• UV laser 355 nm (Molecular Machines & Industries)
• Donor Filterset (Ex: 455/30, DM: 495LP) (Chroma Technology)
• Acceptor Filterset (Ex: 530/20, DM: 585LP) (Chroma Technology)
• Dual View FRET Filterset (DM: 585LP, Em1: 515/30, Em2: 625/30) (Chroma Technology)
• Emission/Excitation filter cube for 25mm filters (Cairn Research, cat # P290/000/200)
• Optosplit II LS Image Splitter (Cairn Research, cat # P280/210/MLS)
• ORCA-Flash 4.0 LT Digital CMOS camera (Hamamatsu, cat # C11440-42U)
• MATLAB Software (Mathworks) (download Biosensors 2.1 package from http://lccb.hms. harvard.edu/software.html)

## PROCEDURE

**Figure 1.**
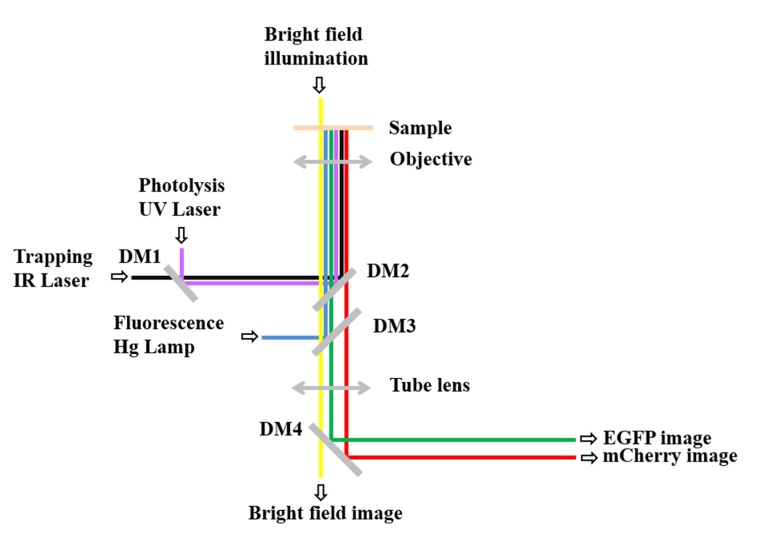
**Schematic representation of the optical manipulation and FRET imaging setup.** The InfraRed (IR) laser used for trapping micro-particles is aligned with the UltraViolet (UV) laser used for photolysis and then they are both directed into the objective pupil (60 ×, 1.2 NA). The sample is illuminated both by a white light source and a Hg fluorescence lamp. The light coming from the excited sample is then separated into the two emissions of the Donor and Acceptor fluorophores for FRET imaging by the beam splitter (DM4, 585 LP) and directed onto the two halves of the camera sensor.

**Figure 2.**
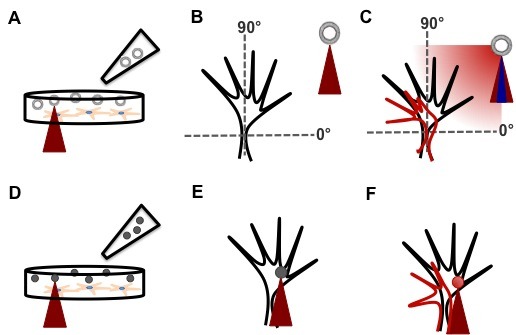
**Schematic representation of the local stimulation protocols. A.** Pipette 5–8 µl of solution containing lipid vesicles into the imaging chamber, wait for them to sink and trap one of them by focusing the IR laser in the center of the vesicle. **B.** Gently move the trapped lipid vesicle in the proximity (10–20 µm) of the cell compartment (growth cone (black line) in this case) to stimulate, with an angle between 0–90^°^. **C.** Break the lipid vesicle to release its content and locally deliver the protein to the growth cone. In this case, a repulsive cue triggers collapse and retraction from the source (red line depicts putative morphological change after stimulation). **D.** Same as in (**A**), but pipette 5–8 µl of coated beads into the chamber. **E.** Gently move the trapped bead over the cell compartment (central part of the growth cone (black line) in this case) to stimulate and leave it there for the chosen amount of time. **F.** In this case, a repulsive cue mediates collapse and retraction of the stimulated region (red line depicts putative morphological change after stimulation).

1. Preparation of coverslips
**NOTES**: The coverslip preparation step is meant to clean, sterilize and coat the surface of glass coverslips for cell culture.
  1.1. Wash glass coverslips overnight in 0.5 N HCl in a dedicated glass jar or beaker.
  1.2. Remove the coverslips from HCl and wash twice in deionized water, then put them in 100% ethanol for 1.5 h.
  1.3. Separate the coverslips, put them in a glass petri dish and sterilize them in a stove for 3 h at 180^°^C.
  1.4. Place the coverslips in a 12-well plate and coat them with 2 µg/cm^2^ of laminin for 2–3 h.
  1.5. Rinse them twice with 1 ml of pre-warmed cell culture medium. Leave the coverslips in 1 ml of pre-warmed medium.
**NOTES**: Grow and maintain NG108-15 cell line following standard protocols. All cell incubations are performed at 37^°^C, 5% CO_2_ incubator. Equipment and reagents coming into contact with cells must be sterile.
2. Cell transfection
**NOTES**: This protocol has been optimized for the transfection of Cdc42 and RhoA inter-molecular FRET probes developed by Murakoshi *et al*. [19] using Metafectene Easy + reagent.
  2.1. Seed 0.2 × 10^5^ cells into the coverslips in DMEM medium supplemented as described above and leave them in the incubator for 24 h.
  2.2. Prepare Buffer Solution 1 × in sterile H_2_O. Calculate 100 µl of buffer solution for each coverslip to be transfected.
  2.3. Add 1 µg of total plasmid DNA for each coverslip to be transfected.
  2.4. Put 2.5 µl of transfection reagent in each tube and mix gently. Remember to vortex the reagent before adding it to the DNA-buffer solution.
  2.5. After 15 min incubation at room temperature (RT), add the transfection mixture dropwise to the cells. Leave them in the incubator for 18–24 h to ensure complete protein expression.
**NOTES**: (Step 2.3): The inter-molecular FRET biosensors are composed of two different proteins which DNA must be added to a total of 1 µg (0.5 + 0.5) to the transfection solution. Apart from the experiment samples, at least two samples must be transfected with 1 µg of each single probe for later imaging as control and off-line bleedthrough and cross-talk corrections (Steps 5–6).
3. Beads functionalization
**NOTES**: This protocol can be used for any protein to couple and any bead dimension, provided that the beads are functionalized with carboxylic (-COOH) groups.
  3.1. Dilute 3 µl of beads stock solution (diluted at 50 mg/ml) in 1.8 ml PBS.
  3.2. Pipette 60 µl of the diluted beads in a polypropylene micro-centrifuge tube.
  3.3. Centrifuge for 10 min at 1000 × g.
  3.4. Discard the supernatant and suspend the pellet in 100 µl of Coupling Buffer.
  3.5. Centrifuge for 10 min at 1000 × g.
  3.6. Discard the supernatant and suspend the pellet in 50 µl of Coupling Buffer.
  3.7. Prepare a 200 mg/ml 1-Ethyl-3-(3-dimethylaminopropyl)carbodiimide (EDAC) solution in Coupling Buffer and add 10 µl of this solution to the beads suspension.
  3.8. Mix gently and leave to rest at RT for 1 h.
  3.9. Add the protein of choice at saturation. For the right amount of protein calculate 20–500 ng of protein for each mg of particles.
  3.10. Leave to rest for 1 h at RT with gentle mixing.
  3.11. Centrifuge for 10 min at 1000 × g, then remove the supernatant and suspend the pellet in 400 µl of Wash/Storage Buffer. Repeat this washing procedure twice.
  3.12. Store the particles at 4^°^C for up to two weeks.
**NOTES**: (Step 3.11): Keep the supernatant for the calculation of the amount of protein linked to the bead with the BCA protein assay.
**NOTES**: This protocol is optimized for the BioMag Plus Carboxyl Coupling Kit.
4. Lipid vesicles preparation
**NOTES**: This protocol is meant to produce mono- or multi-layered lipid vesicles with a diameter varying between 1–10 µm.
  4.1. Prepare a 6 ml solution of chloroform:methanol in proportion 2:1 (v/v).
  4.2. Add the chloroform:methanol solution to the liposome kit vial to dissolve the lipid powder. Saturate the vial with N_2_ (lipid vials can be stored at −20^°^C until expiration date).
  4.3. Put 50 µl of the lipid solution in one or more glass vials and proceed to the lipid desiccation in vacuum (18 h). This step results in semi-transparent lipid films on the sides of the glass vials.
  4.4. Remove the glass vials from the oven and immediately saturate with N_2_. Proceed with the lipid film rehydration preparing a solution (Solution A) with 20 µl PBS + 5 µl Sucrose at 1 M concentration for each vial to rehydrate.
  4.5. Prepare a 25 µl solution in sterile PBS containing the protein of choice at the desired concentration (Solution B) for each lipid film to rehydrate. We used 1 µM Semaphorin-3A final concentration [17,19].
  4.6. Mix well together Solution A and Solution B (50 µl in total). Add gently the 50µl rehydrating solution to each glass vial containing the lipid film.
  4.7. Vortex for about 1 min and leave them overnight at 4^°^C.
  4.8. The day after, prepare a 100 mM Glucose washing solution in PBS.
  4.9. Add 500 µl of washing solution in a 500 µl micro-centrifuge tube.
  4.10. Add gently 10 µl of the lipid vesicles solution previously prepared on the surface.
  4.11. Pellet the lipid vesicles in a table top centrifuge for 3 min at 1500 × g. Add 500 µl of washing solution and repeat this process again.
  4.12. Resuspend the final pellet in 50 µl of washing solution. Add part of the lipid vesicles solution directly in the plate to perform the local stimulation experiments. Store in the fridge the remaining solution to use for further experiments.
**TIPS**: (Step 4.3–4.4): The lipid desiccation in the vacuum oven must be performed with the vials without caps. Do not prepare more than 5–6 vials each time because prolonged air contact may oxidize the lipids and impair the vesicle efficacy.
**TIPS**: (Step 4.11): Be cautious when removing the supernatant because the pellet is not visible and very delicate.
**NOTES**: These lipid vesicles ought to be used in experiments only in the day of production, since they tend to deteriorate quickly.
5. Local stimulation and FRET imaging
**NOTES**: For these experiments an inverted optical microscope equipped with an InfraRed (IR) optical tweezers and an UltraViolet (UV) microsurgery laser for optical manipulation of the vectors loaded with SemA3 is necessary. The setup includes also a fluorescence imaging path adapted for FRET imaging: Donor and raw (r)FRET images are recorded simultaneously on the two halves of the CMOS sensor (**Fig.1**). Two filtersets (1- Donor excitation filter + dichroic, 2- Acceptor excitation + dichroic) are mounted in the fluorescence filter wheel of the microscope, and an optical splitter with emission filters for Donor (D) and Acceptor (A) is mounted at the exit port of the microscope to record two channels.
  5.1. Prepare the Ringer’s solution at pH 7.4.
  5.2. Carefully mount the coverslip with cells into the recording chamber, fill it with Ringer’s solution and set the chamber in the temperature controlled stage (37^°^C) of the microscope.
  5.3. Focus the objective on the cells seeking for transfected cells. Choose an isolated cell with neurite-like protrusions and growth cones.
**NOTE**: An optimal transfection efficiency is around 40%–60%.
  5.4. Drop 5–8 µl of lipid vesicles or beads in the recording chamber and then turn the IR trapping laser on.
  5.5. To stimulate with lipid vesicles adopt the following procedure.
    5.5.1. Trap a lipid vesicle and gently move the stage and the objective to position the trapped vesicle in the proximity (10–20 µm) of the cell compartment to be stimulated. (**Fig. 2A-2C**).
    5.5.2. Using D excitation acquire rFRET and D fluorescence images for 90 s, to assess its spontaneous activity and dynamics.
    5.5.3. Acquire a video of the lipid vesicle photolysis using UV laser nanosecond pulses. Use Differential Interference Contrast (DIC) for imaging.
    5.5.4. Switch off the UV, IR laser beams, and the DIC imaging. Start acquisition of rFRET and D fluorescence images.
  5.6. To stimulate with coated beads adopt the following procedure.
    5.6.1. Record rFRET and D fluorescence images of the cell for 90 s to assess its spontaneous activity and dynamics.
    5.6.2. Trap a bead and move the stage and the objective to position the trapped bead in contact with the cell membrane at the desired site of stimulation. (**Fig. 2D-2F**).
    5.6.3. Keep the trap ON for the time of the stimulation (30 s in this protocol), acquiring a video in DIC. Then turn the IR laser OFF.
    5.6.4. Switch OFF the IR laser and the DIC imaging and acquire rFRET and D fluorescence images.
**NOTES**: The experimental observation after stimulation lasts 15 min in our experiments, acquiring fluorescence images continuously with exposure time 1 s, binning × 4.
  5.7. Generate the control images required to correct the raw data in the image processing step (step 6).
    5.7.1. Prepare and mount a fresh coverslip without cells, just with identical media and mounting conditions as that used for cells. Acquire minimum 20 images for both channels, using D excitation filterset. The resulting image set for each will be averaged later in the image processing for shading (uneven illumination) correction. Switch the fluorescence lamp off and acquire another set of minimum 20 images for dark current noise correction.
    5.7.2. Mount into the recording chamber a control coverslip with cells transfected only with the D sensor molecule. Using D excitation, acquire several (3–5) images of transfected cells in both D and A channels. These images will be used later to calculate the bleedthrough correction coefficient.
    5.7.3. Mount into the recording chamber a control coverslip with cells transfected only with the A sensor molecule. Acquire several (3–5) images of transfected cells in A channel using D and A excitation, respectively. These images will be used to calculate the cross-talk correction coefficient.
**NOTES**: (Step 5.7.1): Averaging over a large number of images is important to reduce the stochastic camera noise present in each image frame.
**CAUTION**: This protocol involves the use of class 3b lasers and will require proper training and safety guidelines to be followed.
6. Image processing and ratio calculation.
**NOTES**: The goal of this step is to calculate the ratio image indicating the activity of the GTPases, starting from the rFRET and D images. This requires that any potential differences between these images be removed prior to calculating the ratio image. Therefore, several corrections, which are specific to the imaging system used to collect the raw data, are required. These corrections are termed: 1) dark current and shading corrections to ensure that the measured spatial variations in image intensities are accurate within each image and comparable across the different image channels, 2) background subtraction and photobleaching corrections to ensure that the measured intensities are comparable over time and across experiments at the whole image level and finally, 3) spectral overlap and imperfect spectral filters cause bleedthrough and cross-talk between D and A channels, which should be corrected to produce fully independent raw FRET image (rFRET) and D images.
If not otherwise mentioned, the image processing in this protocol was performed using the Biosensors 2.1. Matlab package (Danuser laboratory: http://lccb.hms. harvard.edu/software.html) of which flowchart is described in reference [20].
  6.1. Split and align the D and rFRET images recorded on the two halves of the CMOS sensor during the experiment (step 5). We used a custom Matlab code here.
  6.2. Correct the D and rFRET images for the dark current noise and shading, using the corresponding control images collected at step 5.7.1.
  6.3. Image segmentation for D and rFRET to find the cell edge, create a background mask and subtract background.
  6.4. Calculate the bleedthrough and cross-talk correction coefficients using the control images acquired at step 5.7.2 and 5.7.3 respectively. For each image select at least 3 regions of interest (ROIs) of cells and calculate the average intensity. Use ImageJ or Matlab Image Processing toolbox.
    6.4.1. The bleedthrough coefficient, B, indicates how much light emitted by D bleeds through the A (FRET) channel. It is calculated using D and A images from control cells expressing only D, upon D excitation: *B* = *I*^A^_D_/ *I*^D^_D_, where *I*^A^_D_ is the intensity in A channel and *I*^D^_D_ the intensity in the D chanel, with D excitation.
    6.4.2. The cross-talk coefficient, C, indicates how much the D excitation induces A emission, due to the overlap of the D and A excitation spectra. It is calculated using D and A images from control cells expressing only A, upon D and A excitation: *C* = *I*^A^_A_/ *I*^D^_A,_ where *I*^A^_A_ is the intensity in A channel and *I*^D^_A_ the intensity in the D channel, with D excitation.
**NOTES**: In the case of inter-molecular (dual chain) FRET biosensor such as the biosensors we used in this protocol, the Ratio calculation is defined by the following equation [7]:
(Eqn. 1)
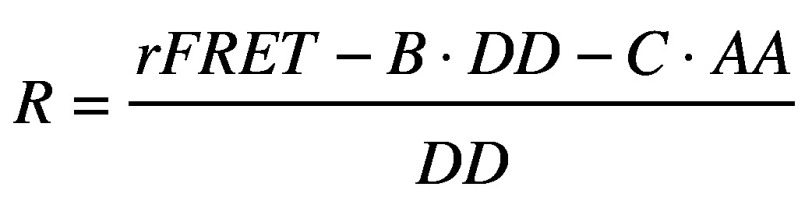

where, *R* is the Ratio, *rFRET* is the raw FRET image, *DD* is the Donor image (intensity in *D* channel upon *D* excitation), *AA* is the Acceptor image (intensity in *A* upon *A* excitation), *B* and *C* the correction coefficients. Notice that three images are usually required in the above equation: *rFRET*, *DD* and *AA*. However, if the cross-talk correction coefficient *C*, is negligibly small, the weighted contribution of the Acceptor *AA*, can be neglected and the equation becomes:
(Eqn. 2)
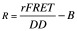

In this case the acquisition of the third image can be avoided, having at least two experimental advantages: reduce the cell exposure to light and avoid mismatching between the third image *AA*, which should be acquired at a different moment of time, with the *rFRET* and *DD* images, which are acquired simultaneously. In order to reduce the cross-talk, we chose the *D* excitation filter (445/30) centered on the minimum of the Acceptor A excitation spectrum. The measured correction coefficient C = 0.018 (mean value), which we considered negligibly small. Equation (2) is equivalent with the ratiometric formula for intra-molecular biosensors, since the same constant B, is subtracted always for each image pair, rFRET-DD. Choosing properly the Acceptor emission filter one can reduce also the bleedthrough of the light emitted by Donor through the Acceptor channel. The measured coefficient in our setup is, *B* = 0.055 (mean value).
  6.5 Correct photobleaching and calculate the Ratio images R using the corrected images with Biosensors 2.1. The final result is a set of images in tiff format, indicating the spatio-temporal activation of the RhoGTPase.

## ANTICIPATED RESULTS

Local stimulation based on optically manipulated carriers containing active molecules is a flexible technique easy to integrate with the optical microscope for live cell analysis. Different imaging techniques can thus be used in parallel with optical manipulation, as phase contrast, fluorescence, or FRET microscopy. Micro-carriers are generally employed since it is easier to observe and monitor them by bright field imaging inside the sample chamber. Both carriers used in this work (beads and lipid vesicles) can deliver any type of active proteins, which makes them practical for a very wide range of applications.

By using the method described herein, we were able to stimulate neuronal growth cones by mimicking the natural stimuli that lead to neuronal cell migration. We observed morphological rearrangements within the growth cones upon local Sema3A release.

**Figure 3.**
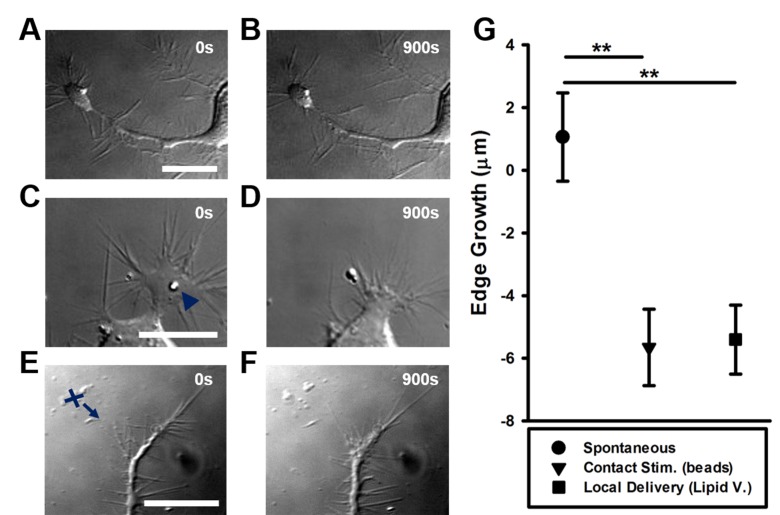
**Representative results of a repulsive local stimulation experiment on NG108-15 cells. A** and **B.** DIC images of a cell undergoing spontaneous motion at 0 s (**A**) and after 900 s from the acquisition (**B**). **C** and **D.** DIC images of a cell during local repulsive stimulation with Sema3A-coated beads (the blue arrowhead indicates the bead position). **C.** Stimulated cell at the beginning of the stimulation. **D.** Stimulated cell after 900 s from the stimulation. **E** and **F.** DIC images of a cell during local delivery of 1 µM of Sema3A (the blue cross and arrow indicate the position of the lipid vesicle. **E.** Stimulated cell at the breaking of the lipid vesicle. **F.** Stimulated cell after 900 s from the breaking. **G.** Plot of the mean edge growth of cells undergoing spontaneous motion (circle), and cells stimulated locally with a repulsive stimulus with beads (triangle) and lipid vesicles (square). There is a significant difference between the non-stimulated cells ((*n* = 12) mean edge growth: 1.1 ± 1.4 µm) and the cells stimulated with beads ((*n* = 6) mean edge growth: −5.7 ± 1.1 µm) and lipid vesicles ((*n* = 12) mean edge growth: −5.4 ± 1.0 µm). Scale bars in (**A**), (**B**) and (**C**): 20 µm. ^**^P< 0.01.

**Figure 3** shows an example of the morphological changes occurred in NG108-15 cells upon Sema3A local stimulation with beads (**Fig. 3C** and **3D**) and lipid vesicles (**Fig. 3E** and **3F**) in comparison with a cell that is not stimulated (**Fig. 3A** and **3B**). The plot of the mean edge growth of stimulated cells shows that both local stimulation methods are effective ways to trigger growth cone collapse and retraction, since there is no significant difference in the morphological response to either stimulation methods (**Fig. 3G**).

The combination of FRET imaging with local stimulation allows the monitoring of the spatial and temporal activation of signaling molecules (*e.g.* RhoGTPases) in response to local extracellular stimuli (*e.g.* guidance cues). The dynamics of the ratio between Donor and FRET channels reflect the spatio-temporal activation of the proteins studied.

**Figure 4.**
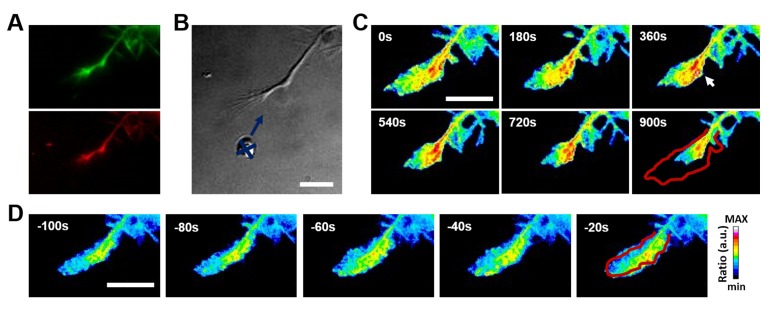
**Representative results of FRET imaging of RhoA after local repulsive stimulation. A.** Example images of EGFP (green) and mCherry (red) fluorescence channels that are separated and corrected off-line to obtain the final ratiometric video (mCherry/EGFP). **B.** DIC image of the growth cone undergoing local delivery of 1 µM Semaphorin-3A (The blue cross and arrow indicate the position of the lipid vesicle). **C.** Ratiometric FRET live imaging of a representative cell transfected with RhoA inter-molecular probe. The insets show a time series (1 frame every 3 min) for 900 s after the stimulation. The white arrow highlights the increase of FRET signal in the center of the growth cone subsequent to its retraction. The red line shows the initial edge profile. **D.** Ratiometric FRET live imaging of the cell in (**C**) before the local stimulation. The insets show a time serie (1 frame every 20 s) from 100s before the stimulation. The red line shows the initial edge profile. The intensity scale for the ratio on the right is expressed in arbitrary units (a.u.) and applies to all the images. Scale bars in (**B**), (**C**) and (**D**): 20 µm.

**Table 1.** Troubleshooting.

Step	Problems	Causes	Suggestions
2	Poor transfection efficiency	DNA quantity is too low	Modify the transfection reagent/DNA ratio by adding more DNA to the mixture
2	Low cell viability	• DNA quantity is too high • Incubation time is too high	• Decrease the amount of transfection solution and DNA added to the cells • Decrease the incubation time NOTE: Longer incubation times may result in higher efficiency but higher toxicity also
3	Low BCA result	Low protein linked to the bead	Check that the amount of protein added is at saturation for the linker reaction to successfully take place
4	No lipid vesicles detected in the solution	• No lipid vesicles present • Wrong molarity of the solutions	• Be careful in all the washing procedures • Check the correct molarity of the glucose and sucrose solutions
5	No fluorescence intensity is detected in the FRET channel	FRET signal is very low	Increase the exposure time or the light intensity; this may bring to excessive photobleaching or phototoxicity, adjust these parameters to optimize the image acquisition
5	Lipid vesicles and beads cannot be trapped	• IR laser is out of focus • IR laser power is too low	• Check the IR laser focus and align the beam • Increase the laser power
5	Lipid vesicles cannot be broken	UV and IR laser are not overlapping	Check the UV and IR laser relative position and focus and align the beam
6	Biosensors 2.1 package doesn't run	MATLAB version does not support it	This package works on MATLAB 2012b or below

**Figure 5.**
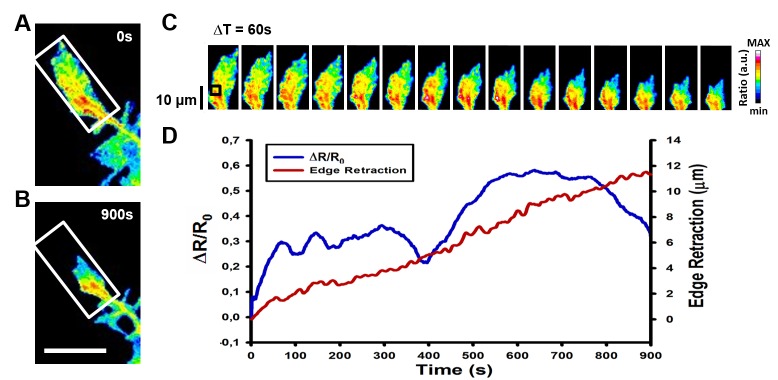
**Further analysis of FRET ratiometric images. A** and **B.** Example FRET ratiometric images of the cell in **Fig. 4** taken at 0 s (**A**) and 900 s (**B**) from the stimulation. The white box in both images refers to the area used in the image montage in (**C**). Scale bar in **Fig. 5B** refers to **Fig. 5A** and **5B**: 20 µm. **C.** Image montage of the region highlighted by the white box in (**A**) and (**B**). Images are taken every 60 s. The intensity scale on the right refers to **Fig. 5A**, **5B** and **5C**. **D.** Plot of the RhoA activity, normalized by the initial ratio value (ΔR/R_0_), in the edge region highlighted by the black square in (**C**) *vs*. edge retraction that shows the increase in RhoA activity as the growth cone retracts.

**Figure 4** shows that Sema3A repulsive stimulus triggers an activation of RhoA in both the center of the growth cone that diffuses to the leading edge and in filopodia causing growth cone collapse and retraction. The dynamic changes can be further investigated by using image montages (**Fig. 5A-5C**) or by performing Area Intensity Analysis to plot the activity changes versus the leading edge dynamics (**Fig. 5D**).

In summary, this methodology can be used to study the dynamic growth cone rearrangement in response to different guidance cues, and has been recently defined as a very promising method for guidance cues delivery [21,22]. Since a great variety of FRET probes have been created to study the dynamics of many proteins [23-26], this combination provides a very useful tool for investigating intra-cellular signaling pathways at the basis of major cell dynamics.

However, the development of new vectors - for which the molecules release can be timely tuned - and of more sensitive FRET probes will represent important optimizations of this technique in the future to broaden even more its potential applications.

## TROUBLESHOOTING

**Table 1** presents issues and problems that are most likely to be encountered during this procedure and some hints and solutions to solve them. The setup configuration, alongside IR and UV relative alignment are the most delicate and should be always checked and addressed carefully, since a misalignment between the two beams brings to impossibility in trapping the beads and breaking the lipid vesicles.

## Abbreviations used

BCA: Bicinchoninic Acid
BDNF: brain-derived neurotrophic factor
DIC: differential interference contrast
DMEM: Dulbecco's modified Eagle medium
FBS: fetal bovine serum
FRET: Förster resonance energy transfer
GDP: g-DI-Phosphate
GTP: guanosine triphosphate
IR: InfraRed
PBS: phosphate buffered saline
RT: room temperature
UV: ultraviolet
